# IFNα and β Mediated JCPyV Suppression through C/EBPβ-LIP Isoform

**DOI:** 10.3390/v13101937

**Published:** 2021-09-26

**Authors:** Dana May, Anna Bellizzi, Workineh Kassa, John M. Cipriaso, Maurizio Caocci, Hassen S. Wollebo

**Affiliations:** 1 Department of Neuroscience, Center for Neurovirology—Lewis Katz School of Medicine at Temple University, 3500 N. Broad Street, Philadelphia, PA 19140, USA; tul10237@temple.edu (D.M.); tuf81370@temple.edu (A.B.); john.cipriaso@temple.edu (J.M.C.); tuh20265@temple.edu (M.C.); 2 Mayo Clinic Hospital and Health Care, 200 First St. S.W., Rochester, MN 55905, USA; etgold4@gmail.com

**Keywords:** progressive multifocal leukoencephalopathy, polyomavirus JC, interferonα and β signaling, C/EBPβ-LIP, CRISPR Cas9 STAT-1 knockout

## Abstract

Polyomavirus JC (JCPyV) causes the demyelinating disease progressive multifocal leukoencephalopathy (PML). JCPyV infection is very common in childhood and, under conditions of severe immunosuppression, JCPyV may reactivate to cause PML. JC viral proteins expression is regulated by the JCPyV non-coding control region (NCCR), which contains binding sites for cellular transcriptional factors which regulate JCPyV transcription. Our earlier studies suggest that JCPyV reactivation occurs within glial cells due to cytokines such as TNF-α which stimulate viral gene expression. In this study, we examined interferon-α (IFNα) or β (IFNβ) which have a negative effect on JCPyV transcriptional regulation. We also showed that these interferons induce the endogenous liver inhibitory protein (LIP), an isoform of CAAT/enhancer binding protein beta (C/EBPβ). Treatment of glial cell line with interferons increases the endogenous level of C/EBPβ-LIP. Furthermore, we showed that the negative regulatory role of the interferons in JCPyV early and late transcription and viral replication is more pronounced in the presence of C/EBPβ-LIP. Knockdown of C/EBPβ-LIP by shRNA reverse the inhibitory effect on JCPyV viral replication. Therefore, IFNα and IFNβ negatively regulate JCPyV through induction of C/EBPβ-LIP, which together with other cellular transcriptional factors may control the balance between JCPyV latency and activation.

## 1. Introduction

### 1.1. The Human Polyomavirus JC

Neurotropic Polyomavirus JC (JCPyV) causes progressive multifocal leukoencephalopathy (PML), a severe and often lethal demyelinating disease of the central nervous system (CNS). In PML, JCPyV actively replicates in the glial cells of the CNS (oligodendrocytes and astrocytes) causing lytic cell death and leading to the formation of multiple expanding lesions of demyelination in the brain and worsening neurological dysfunction [1,2]. Despite the occurrence of JCPyV infection in the worldwide population being very common, PML occurs infrequently, and it is almost always found in individuals with severe immune system dysfunction, particularly HIV-1/AIDS, where PML remains a complication even after the prevalent use of combination antiretroviral therapy [3]. Most people are infected by the virus early in life, but the immune system efficaciously controls JCPyV replication. However, the virus is able to persist in a latent asymptomatic state, which is only poorly understood [4,5]. Rarely and under condition of severe immune impairment, JCPyV reactivates to replicate in the glia of the brain leading to PML and understanding the molecular mechanism by which this occurs is very important for JCPyV biology and the pathogenesis of PML.

JCPyV is a small non-enveloped human Polyomavirus with a circular double-stranded DNA genome, 5.1 kb in length, composed of three regions: one bidirectional regulatory element and two protein coding regions that produce early and late transcript [6,7]. The bidirectional regulatory element, or non-coding control region (NCCR), contains the promoter and enhancer elements for the early and late coding regions and the origin of viral replication. The products of the early transcript are viral regulatory proteins such as Large T antigen (TAg), small t antigen and T’ proteins that are required for viral replication and *trans* activation of the late promoter by TAg. The late region encodes structural capsid proteins VP1, VP2 and VP3 and small regulatory protein called Agno [7]. Our previous studies showed that the transition from the inactive persistent state to active replication (viral reactivation) initiates PML and it is thought to involve signaling events in glial cells triggered by cytokines such as TNF-α, IL-1β, IL-6 and TGF-β [8,9] acting through transcription factors including NF-κB, C/EBPβ and NFAT4 [10,11,12]. In contrast, interferons have been reported to negatively regulate JCPyV [13,14].

### 1.2. C/EBPβ-LIP Isoform

Members of the CAAT/enhancer binding proteins (C/EBPs) family play a pivotal role in several cellular processes such as growth and differentiation, metabolism, immune and inflammatory processes as well as various responses to diseases [15]. The C/EBPs family consists of six DNA binding proteins, α, β, γ, δ, ε, and ζ, all of which share greater than 90% of their sequence identity. The C-terminal of their amino acid residues consists of a region referred to as the ZIP domain; within this domain exists a basic amino acid-rich DNA binding region followed by a dimerization motif also known as the “leucine zipper” which along with the basic domain is essential to DNA binding [10,15].

The transcription factor C/EBPβ has three isoforms: full length (FL), liver activating protein (LAP), and liver inhibitory protein (LIP). These transcription factors can bind to the CAAT enhancer site of many gene promoters to downregulate or upregulate expression. In the case of JCPyV, C/EBPβ-LIP binds to the NCCR and acts as a negative regulator to downregulate JCPyV early and late protein transcription [10]. A previous study conducted by Bellizzi et al. showed that C/EBPβ-LIP overexpression has been associated with large (T) antigen downregulation and degradation [16]. Additional studies examined C/EBPβ’s effect on HIV-1 replication in monocytes and macrophages [17,18]. Briefly, Tesmer et al. reported in Jurkat cell line that the nuclear factor for IL-6 expression (NF-IL6) is a member of a C/EBP family and its binding site in the negative regulatory element type 1 (NRE1) region of HIV-1 long terminal repeat (LTR) acts as a repressor of HIV-1 transcription [17]. Moreover, Honda et al. have also shown that repression of the HIV-1 LTR required intact C/EBP sites and THP-1 cell-derived macrophages treated with INFβ induce C/EBPβ-LIP and coincidentally repressed HIV-1 LTR transcription [18].

### 1.3. Type I IFNs and Antiviral Responses

Type I interferons (IFNs) are the principal cytokines involved in antiviral responses and include interferon-α (IFNα) or β (IFNβ) among other members [19,20]. Their essential role in eliciting innate and adaptive immune responses to combat viral replication and infection has been well established in *ifnar1^−/−^* mice studies [21,22]. Additionally, IFNs help to control viral infection in the CNS by inducing cell junction tightening in microvasculature endothelial cells to restrict BBB permeability, which has also been noted to occur in astrocytes, and neuro-invasion [23,24].

IFNβ binding to IFNα receptor 1 (IFNAR1) and IFNα receptor 2 (IFNAR2) activates the JAK-STAT signaling pathway which leads to the formation of phosphorylated STAT1 and STAT2 heterodimer complex. This complex induces the transcription factor ISGF3 which binds to IFN-stimulated response elements (ISREs) to promote the expression of IFN-stimulated genes (ISGs) which then lead to the production of antiviral effectors [25]. Through these signaling pathways, IFNs control viral replication thus viral spread through the induction of an antiviral state in infected and uninfected cells, which is done by the promotion of antiviral effector ISGs within infected and neighboring uninfected cells [26]. In addition to the JAK-STAT pathway, interferons have shown to exert their antiviral activity by modulating the function of some transcriptional factors such as C/EBPβ families [15,27]. Honda et al. have reported that the treatment of macrophages with IFNβ induced a dominant-negative C/EBP transcription factor which repressed the HIV-1 LTR promoter. These findings suggest that one mechanism of viral replication inhibition is by type I IFNs acting as a transcriptional repressor [18].

In this study, we examine the effect of type 1 IFNs specifically α and β on JCPyV viral replication which proved to have a negative effect on JCPyV transcriptional regulation. Furthermore, our results also show that treatments of IFNα and β increased endogenous levels of C/EBPβ-LIP in a time-dependent manner. Additionally, we show that the negative regulatory role of IFNα and IFNβ in JCPyV early and late transcription is more pronounced in the presence of C/EBPβ-LIP. Therefore, C/EBPβ-LIP negatively regulates JCPyV early and late promoter activity, which together with other cellular transcriptional factors may control the balance between JCPyV latency and activation leading to PML.

## 2. Materials and Methods

### 2.1. Cell Culture and Plasmids

Human TC620 oligodendroglioma cells line was obtained from Dr. Bassel Sawaya [28]. SVGA are cell line derived from primary human fetal glial cells transformed by origin-defective SV40 that expresses SV40 T-Ag and were purchased from ATCC (ATCC CRL-8621, Manassas, VA, USA). Both the cell were maintained in Dulbecco’s Modified Eagle’s Medium (DMEM, Mediatech INC, Manassas, VA, USA) supplemented with 10% fetal bovine serum (FBS) and 25 μg/mL of Gentamicin (stock solution 50 mg/mL from Mediatech INC, Manassas, VA, USA) in an incubator at constant temperature of 37 °C and constant injection of 5% CO_2_. The reporter constructs JCPyV_Early_-LUC (JCPyVE) and JCPyV_Late_-LUC (JCPyVL) derived from the plasmid pGL3 (Promega, Madison, WI, USA) have been previously described [8] and they contained the full-length NCCR of JCPyV Mad-1 strain (NCBI Reference Sequence: NC_001699.1) cloned into the *SmaI* site immediately upstream of the luciferase gene in the early and late orientations, respectively. The expression plasmid pCMV-C/EBPβ-LIP (pLIP) was kindly provided by Dr. Ueli Schebler, University of Geneva (Geneva, Switzerland). In this plasmid, the cDNA encoding the liver-enriched transcriptional-inhibitory protein (C/EBPβ-LIP, 20 kDa), an isoform of the CAAT/enhancer binding protein beta (C/EBPβ), has been cloned downstream of the Cytomegalovirus promoter (CMV). The pcDNA6A (pcDNA^TM^6/V5-His A, Invitrogen Life Technologies, Carlsbad, CA, USA) was also used as mock plasmid for the monitoring of transfection effect on cell viability.

### 2.2. Antibodies

The following antibodies were used to perform Western blots: mouse anti-α-tubulin (B7) antibody; mouse anti-GAPDH (6C5) antibody; rabbit anti-STAT1 (E-23) p84/p91 antibody and mouse anti-CEBPβ (H7,sc-7962) antibody were all ordered from(Santa Cruz Biotechnology INC., Santa cruz, CA, USA) and were each stored in 4 °C; anti-VP1 antibody (AB597) was kindly provided by W. Atwood (Brown University, Providence, RI, USA) and was stored in −20 °C; rabbit anti-phospho (Y701)-STAT1 (58D6) antibody and rabbit anti-lamin A/C antibody(clone 4C11) were all ordered from (Cell Signaling Technology, Danvers, MA, USA) and were stored at minus 20 °C; mouse anti-FLAG M2 antibody (Sigma-Aldrich St. Luis, MO, USA) was used to detect FLAG-tag SpCas9 in Western blot assay and it was stored at minus 20 °C. IRDye^®^ goat anti-mouse 800CW, IRDye^®^ goat anti-mouse 680RD and IRDye^®^ goat anti-rabbit 680RD were used as secondary antibodies and purchased from LI-COR, Inc. (Lincoln, NE, USA). The mouse anti-CEBPβ (H7) antibody was also used to perform Immunocytochemistry and Goat Anti-Mouse IgG H&L (FITC) (abcam) was used as FITC conjugated anti-mouse secondary antibody in this assay.

### 2.3. Recombinant Human IFNα and IFNβ Treatment

SVGA cells were cultured at 70% confluency in a 6-well plate and treated with IFNα 50 ng/mL (Millipore Sigma) or IFNβ 100 ng/mL (Millipore Corp, Darmstadt, Germany) for 4, 8, 12, 24, 36 and 48 h. Total cell extract were prepared and analyzed by Western blot.

### 2.4. CRISPR Cas9 Knockout of STAT1

The genomic sequence of human STAT1 gene (NC_000002.12 Homo sapiens chromosome 2, GRCh38.p12 Primary Assembly, REGION: 190968989–191014250) was obtained from NCBI data base and STAT1 open reading frame was determined using the Benchling online platform (www.benchling.com last accessed date 25 September 2021). Two gRNAs targeting the human STAT1 Exon15 and 16 was designed using Benchling CRISPR design tool (www.benchling.com last accessed date 25 September 2021). The gRNA candidates were selected based on the functional domain they target and the highest *on target* cleavage scores (Table 1). A pair of oligonucleotides for each targeting site was designed in forward and reverse orientation as follows: STAT1e15Fw 5′-CAC CGT GCT GGC ACC AGA ACG AAT G-3′ and STAT1e15Rev 5′-AAA CCA TTC GTT CTG GTG CCA GCA C-3′ for exon 15 of STAT1 gene, STAT1e16Fw 5′-CAC CGT GGT TTG GTA ATT GAC CTC G-3′ and STAT1e16Rev 5′-AAA CCG AGG TCA ATT ACC AAA CCA C-3′ for exon 16 of STAT1 gene (Table 1). The annealed oligonucleotides for each gRNAs were phosphorylated and ligated to the linearized vector: each oligonucleotide contains sticky ends for cloning in a tandem gRNAs U6 cassettes in pX333 plasmid (Plasmid #64073 by Addgene) after sequential cutting by *BbsI* and *BsaI* [29]. The same plasmid expresses also the CRISPR endonuclease SpCas9. The ligation mixture was transformed into competent cells and the cloning of gRNAs were confirmed by Sanger sequencing using the oligonucleotides described above as primers.

### 2.5. Production of Clonal Derivatives of SVGA Expressing SpCas9 and STAT1-Specific gRNAs

SVGA cells were co-transfected with pX333 or pX333-derived plasmids expressing the two STAT1-targeting gRNAs (described above) and the plasmid pKLV-U6gRNA (Plasmid #62348 by Addgene: Watertown, MA, USA) containing the puromycin resistance in a ratio of 5:1. Selection was done with 1 μg/mL puromycin, and clones isolated by dilution cloning.

### 2.6. Reverse Transcriptase Reaction

The STAT1 gRNAs expression were assessed on the whole RNA extracted by the RNeasy Mini Kit following the manufactory protocol (QIAGEN, Germantown, MD, USA). The RNA was retro-transcribed in cDNA by the M-MLV reverse transcriptase (Invitrogen Life technologies, Carlsbad, CA, USA) using the primer T404 (5′-AAA AGC ACC GAC TCG GTG CCA C-3′) that anneals on the SpCas9 sgRNA scaffold. The gRNAs specific sequences were amplified by PCR from cDNA using primer T404 as reverse and primers STAT1e15Fw and STAT1e16Fw as forwards (Table 1). The specific PCR products were visualized by electrophoresis on 2% agarose gel.

### 2.7. Analysis of STAT1 Gene Sequence Excision

Since the cleavage of DNA by Cas9 leaves behind characteristic DNA excision, we analyzed the sequence of the STAT1 gene from the SVGA cells stable for STAT1 gRNAs. Total genomic DNA was isolated from cells using a genomic DNA purification kit according to the manufacturer’s instructions (NucleoSpin^®^ Tissue kit (REF 740952-250) by Macherey-Nagel, Duren, Germany) and the regions of the STAT1 gene that had been targeted were amplified by PCR using flanking primers (Table 1). The PCR products were cloned into the TA cloning vector pcR2.1^®^-TOPO TA(REF45-0245) by Invitrogen Life technologies, Carlsbad, CA, USA) and positive colonies were sequenced by Sanger sequencing using the oligonucleotides reported in Table 1 as primers.

### 2.8. Infection/Transfection/shRNA Depletion of pLIP

SVGA cells, which support JCPyV replication, were cultured at 60% confluency in a 6-well plate and infected with JCPyV Mad-1 at MOI of 1 for 2 h. The virus inoculum was removed, and the cell were transduced with Adeno-siRNA-LIP or Adeno-null (control) as we previously described [10]. In experiments to test the effect of C/EBPβ-LIP overexpression in JCPyV replication, cells were transfected with either 2 μg pLIP or pcDNA6A (control) 24 h after viral infection. The transduction of the cells with Adeno vectors and transfection with pLIP continue on days 3 and 4 post infection, respectively. Five days post infection, the efficiency of C/EBPβ-LIP knockdown and its effect on viral replication was evaluated by Western blot. Supernatants were collected to measure the viral load by Q-PCR.

### 2.9. Luciferase Assays

Experiments involving co-transfection of reporter plasmids (JCPyVE and JCPyVL) and expression plasmid (pLIP) were performed as we previously described [8]. Briefly, TC620 cells were transfected with reporter constructs alone (200 ng) or in combination with the expression plasmid. The total amount of transfected DNA was normalized with empty vector DNA (pcDNA6A). When IFNα or β were used, 50 ng/mL of IFNα or 100 ng/mL of IFNβ were added to the culture with a fresh media after six hours of the transfection and the next day the same treatment was repeated. Assay for luciferase was performed as previously described [8]. Briefly, the cells were lysed 48 h after transfection by Cell Culture Lysis Reagent 5X (Promega, Madison, WI, USA). The plates were incubated at room temperature for 5 min on a rocking platform and then placed in −80 °C for at least 1 h. The cells were then scrapped from each well, centrifuged and the supernatant placed into new tubes. In total, 20 μL of protein lysis were added to 50 μL of Luciferase Assay Substrate (Promega, Madison, WI, USA) and the relative light units per seconds (RLS) were read using a DLReady machine manufactured by Zylyx Corp.

### 2.10. Western Blots

Westerns were performed as previously described [30] except that antibody was detected with the LI-COR system. Blots were incubated with IRDye^®^ Li-COR dyes and visualized with an Odyssey^®^ CLx imaging System (LI-COR, Inc., Lincoln, NE, USA) using LI-COR Odyssey software. Band intensities were quantified using the ImageJ software (NIH, Bethesda, MD, USA) and intensities normalized to equal loading control.

### 2.11. Quantitative Real-Time PCR (Q-PCR)

The Q-PCR was performed using Mad-1 JCPyV-specific forward (nt 2392–2412) and reverse (nt 2467–2486) primers, at the final concentrations of 200 and 400 nM, respectively, plus 200 nM of Mad-1 JCPyV-specific probe (nt 2416–2436), fluorescently labeled at the 5′ with HEX probe. Ten microliters of cell culture supernatant were directly analyzed in triplicate in 10 μL reaction mixture containing the above primers and probe in 10X Buffer for *Platinum^®^ Taq* DNA Polymerase (Invitrogen) plus dNTPs mix (Invitrogen). Plasmid DNA containing the Mad-1 JCPyV genome was used to generate a standard curve against which the samples were analyzed using Lightcycler^®^ 480 and LightCycler^®^ 96 software (REF 05815916001 Roche, IN, USA).

### 2.12. Immunocytochemistry (ICC)

ICC was performed as we have described before [31]. Briefly, SVGA cells were cultured at 60% confluency in a 6-well plate and transfected with 2 μg of pLIP or pcDNA6A as well using 3.5 μL/μg DNA of FuGENE^®^ 6 Transfection Reagent (Promega, Madison, WI, USA). The cells were treated either with IFNα or IFNβ on days 1, 2 and 3 post-transfections. Cells were fixed in 4% paraformaldehyde in PBS for 10 min, washed, permeabilized for 5 min with 0.1% Triton X-100, blocked with 5% normal goat serum for 60 min and incubated overnight in 4 °C with mouse anti-CEBPβ (H7) antibody at 1:200 dilution in 0.1% BSA. Cells were then washed, incubated for 2 h with secondary FITC-conjugated goat anti-mouse secondary antibody at a 1:400 dilution, washed, mounted with 4,6-diamidino-2-phenylinole (DAPI)-containing mounting medium (VECTASHIELD, Vector Laboratories Inc., Burlingame, CA, USA), and the ICC images were acquired by BZ-X800 Fluorescence Microscope (KEYENCE Corporation, Itasca, IL, USA) and analyzed using the dedicated software.

#### 2.12.1. MTT Assay

To assess cell viability after any treatment, MTT (3-(4,5-dimethylyhiazol-2-yl)-2,5-diphenyltetrazolium bromide) assay was performed with 5 mg/mL MTT (Amresco, Solon, OH, USA) using an absorbance reader to determine optical density at wavelengths of 570 and 620 nm.

#### 2.12.2. Cell Fractionation

Cells from each well of a 12-well plate were harvested at the corresponding time points (4, 12 and 24 h) after one treatment with IFNs (alpha or beta) and the nuclear and cytoplasmic proteins isolated by NE-PER™ Nuclear and Cytoplasmic Extraction Reagents (Thermo Scientific™, Rockford, IL, USA) following the protocol provided by the manufactory company.

#### 2.12.3. Statistical Analysis

All the values on the graphs are presented as mean and the standard deviations are reported as error bars on the histograms. Student’s *t* test was used to analyze the statistical significance and *p* values < 0.05 were considered statistically significant. The raw data were analyzed by JMP^®^ Pro 15.1.0 statistical analysis software (SAS Institute Inc., SAS Campus Drive, Cary, NC, USA).

## 3. Results

### 3.1. IFNα or IFNβ Induces Endogenous Expression of C/EBPβ-LIP

**Figure 1:**
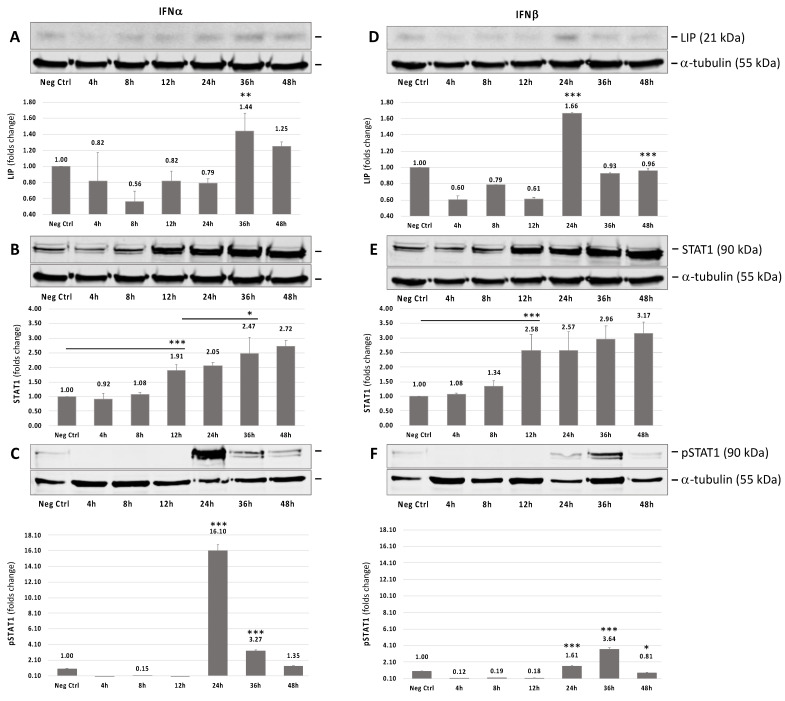
Endogenous expression of c/EBPβ-LIP (LIP) after treatment with IFNα or IFNβ**.** Western blot on protein lysis from SVGA cells collected 4, 8, 12, 24, 36 and 48 h after a single treatment with IFNα (**A**–**C**) or IFNβ (**D**–**F**), respectively, in order to evaluate the endogenous expression of LIP (**A**,**D**). ⍺-tubulin was used as loading control and endogenous expression of STAT1 (**B**,**E**) and its phosphorylated form (**C**,**F**) (phosphoSTAT1 or pSTAT1) were also analyzed. For each panel, a densitometry assessment of LIP and STAT1 was also included, using untreated SVGA cells as negative control. At 36 and 24 h there was a significant increase in endogenous expression of LIP after treatment with IFNα (*p* < 0.01) and IFNβ (*p* < 0.0001), respectively, whereas an important increasing of STAT1 phosphorylation was observed at 24 and 36 h after treatment with IFNα and IFNβ, respectively. Standard deviation bars are depicted on the graph. *p* values < 0.05 were considered statistically significant. Note: * *p* < 0.05; ** *p* < 0.01; *** *p* < 0.0001.

In our earlier studies, we showed that JCPyV NCCR contains a single functional kB element that binds C/EBPβ-LIP and mediates inhibition of JCPyV early and late promoter [10]. Honda et al. [18] report that type 1 interferons induce the inhibitory C/EBPβ-LIP isoform to inhibit the HIV LTR in macrophages. Therefore, we examined the effect of IFNα and IFNβ on the induction of endogenous C/EBPβ-LIP in glia cells (SVGA) (Figure 1). In a time-dependent experiment, we observed a significant increase of LIP expression after 36 (*p* = 0.0011) and 24 h (*p* < 0.0001) of treatment with IFNα (Figure 1A) and IFNβ (Figure 1D), respectively. This expression significantly decreased after 36 h of treatment with IFNβ (*p* < 0.0001) (Figure 1D).

On the other hand, the endogenous expression of STAT1 significantly increased after 12 h of treatment with IFNα (*p* < 0.0001) or IFNβ (*p* = 0.0003) and it slightly increased every 12 h until the end of the experiment, but this trend was not statistically significant (Figure 1B,C, respectively). Moreover, both IFNα and IFNβ induced the phosphorylated form of STAT1 after 24 and 36 h of treatment, respectively (Figure 1C,F, respectively).

We also reported as supplementary data (Supplementary Figure S1) the endogenous expression of the three C/EBPβ isoforms: full length (FL), liver activating protein (LAP), and liver inhibitory protein (LIP). The ratios between the relative expressions of LIP and the isoforms FL and LAP, respectively, were always >1, indicating that LIP was consistently induced after the treatment with the type I IFNs comparing to the respective isoforms.

### 3.2. STAT1-Independent Interferon Signaling

**Figure 2:**
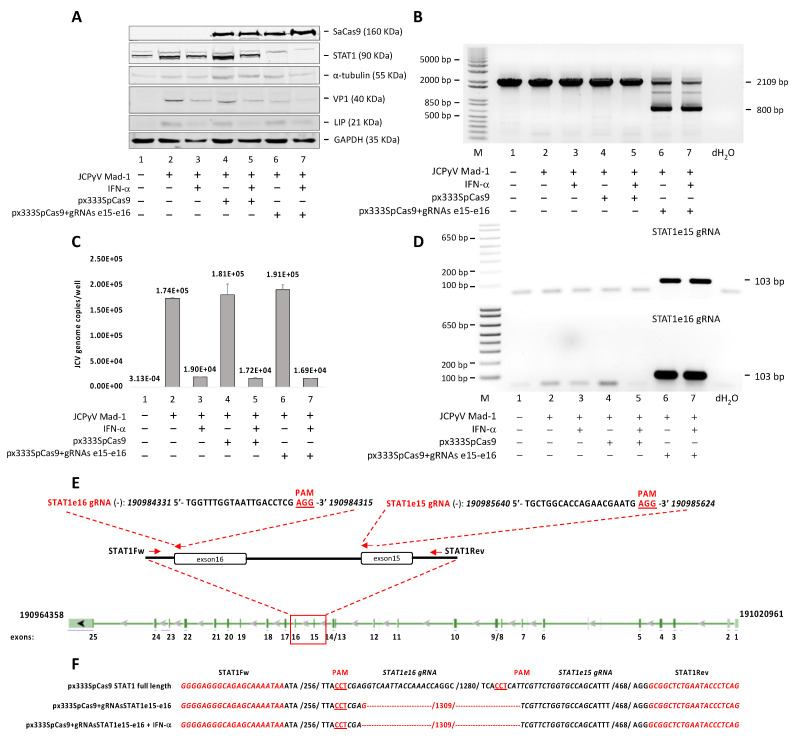
Effect of CRISPR/Cas9 editing of STAT1 on JCPyV replication. SVGA cell line stable for the protein SpCas9 by itself or in combination with two specific single-guide RNAs (gRNAs), STAT1e15 and STAT1e16, targeting the exon 15 and 16 of STAT1, respectively, were produced by the plasmid px333 (see Section 2), infected with the Mad-1 strain of JCPyV at MOI 1 and treated with INF-α (50 ng/mL) every 24 h. The cells were harvested and analyzed at 7 days post-infection (p.i.). (**A**) Fifty micrograms of total cell extract were run on a 10% polyacrylamide SDS gel and analyzed by Western blot for VP1, SpCas9, STAT1 and LIP. Alpha tubulin (α-tubulin) and GAPDH were run as loading controls. (**B**) The STAT1 gene sequence, specifically targeted by the endonuclease SpCas9 in presence of the gRNAs STAT1e15 and STAT1e16, was amplified by PCR, electrophoresed on an agarose gel and visualized with ethidium bromide. (**C**) The JC viral load was also assessed by Q-PCR in the culture supernatant. (**D**) As the expression of the protein SpCas9 was confirmed by Western blot (**A**), the gRNAs expression was assessed by reverse transcription (see Section 2). (**E**) Diagram of the STAT1 gene indicating the positions of PCR primers and gRNAs STAT1e15 and STAT1e16. The PCR primers amplified a sequence of 2109 bp. The endonuclease activity of SpCas9 in the presence of the two gRNAs determined an excision of 1309 bp, generating a new sequence of 800 bp. (**F**) Sequence analysis of PCR products for the STAT1 excision (800 bp) from SVGA cell line stable for the protein SpCas9 and the specific gRNAs STAT1e15 and STAT1e16.

Since the treatment of SVGA cells with IFNα and IFNβ also induces the total and the phosphorylated form of STAT1 (Figure 1B,C,E,F), it was interesting to investigate if this induction has any biological significance on JCPyV replication. To further understand the functional consequence of STAT1 induction and activation by IFNα in JCPyV life cycle, we applied CRISPR Cas9 system to knockout STAT1. As shown in Figure 2A, JCPy infection of STAT1-knockout clonal cell line was suppressed both in the presence and absence of IFNα, indicating that IFNα exerts its anti-JCPyV activity through a STAT1-independent manner. Moreover, STAT1-knockout does not affect the expression of LIP. We notice a slight increase of LIP expression in all the cell lines infected with the virus, but this increase was not significant comparing with the LIP expression under IFNα treatment (Figure 2A). The STAT1 knockout was shown by Western blot (Figure 2A, lanes 6 and 7) and by PCR (Figure 2B). A reduction of viral load in the culture supernatant was also observed by Q-PCR analysis (Figure 2C). The STAT1 gene sequence specifically targeted by the endonuclease SpCas9 in presence of the gRNAs STAT1e15 and STAT1e16 (Figure 2D,E) was amplified by PCR for a final product of 2109 bp. The endonuclease activity of SpCas9 in the presence of the two gRNAs determined an excision of 1309 bp, generating a new sequence of 800 bp (Figure 2B). Finally, the STAT1 excision was confirmed by sequencing (Figure 2F).

### 3.3. Additive Effect of IFNα or IFNβ and C/EBPβ-LIP on Inhibition of JCPyV Early and Late Transcription by Promoter Activity

**Figure 3:**
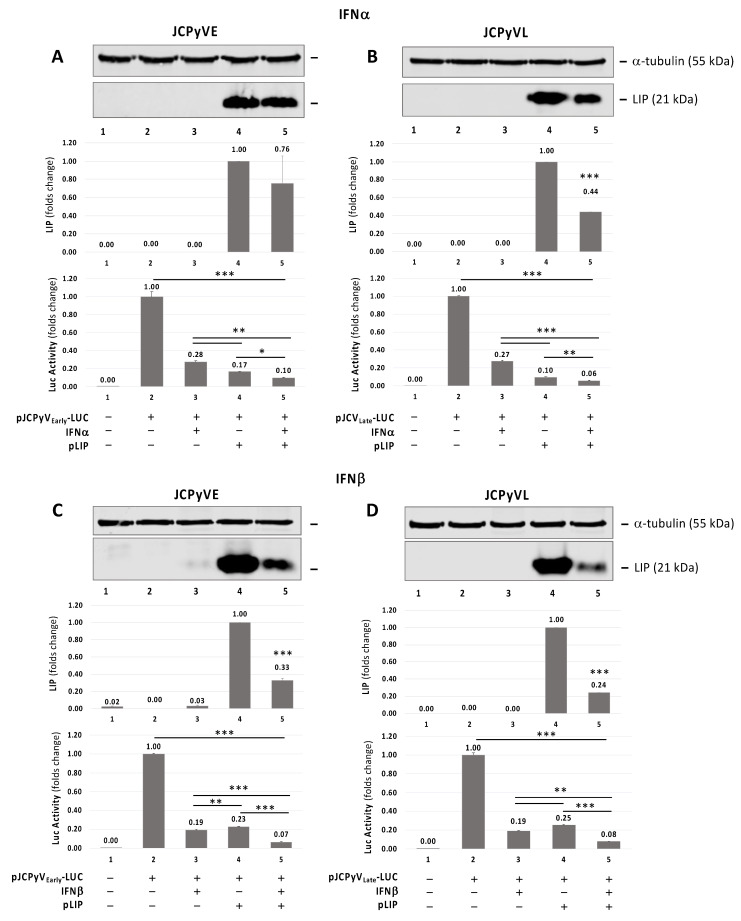
Effect of pCMV-C/EBPβ-LIP (pLIP) expression plasmid and IFNα and IFNβ on JCPyV early and late promoter activity. Western blot on protein lysis from TC620 cells co-transfected with pLIP expression plasmid and JCPyVE (**A**,**C**) or JCPyVL (**B**,**D**) reporter plasmid (containing the full-length NCCR of JCPyV Mad-1 strain cloned immediately upstream the luciferase gene in the early and late orientations, respectively) in presence or absence of IFNα (**A**,**B**) or IFNβ (**C**,**D**) treatment. ⍺-tubulin was used as loading control and overexpression of LIP was analyzed. For each panel, a densitometry assessment of LIP was also included and the luciferase assay results from JCPyVE and JCPyVL, respectively, were reported. A drastic reduction of luciferase signals from JCPyVE and JCPyVL vectors after the IFNα or IFNβ treatment alone was observed. This reduction was significantly pronounced after the co-transfection of the reported plasmids JCPyVE (*p* ≤ 0.05) (**A**) and JCPyVL (*p* < 0.01) (**B**) with the expression plasmid pLIP in presence of IFNα and the co-transfection of the reported plasmids JCPyVE (*p* < 0.0001) (**C**) and JCPyVL (*p* < 0.0001) (**D**) with the expression plasmid pLIP in presence of IFNβ. Note: * *p* < 0.05; ** *p* < 0.01; *** *p* < 0.0001.

To investigate the effect of IFNα or IFNβ and C/EBPβ-LIP overexpression alone or in combination on JCPyV early and late promoter, we used the reporter constructs JCPyV_Early_-LUC (JCPyVE) and JCPyV_Late_-LUC (JCPyVL) containing the full-length NCCR of JCPyV Mad-1 strain cloned immediately upstream the luciferase gene in the early and late orientations, respectively. As shown in Figure 3, treatment of the cells with either IFNα and IFNβ inhibits both early and late transcription as assayed using luciferase reporter plasmid. As we have previously shown [10], C/EBPβ-LIP expression inhibits JCPyV early and late transcription. Additive inhibitory effect of JCPyV early and late transcription was observed when C/EBPβ-LIP-overexpressing cells were treated with either IFNα (Figure 3A,B) or IFNβ (Figure 3C,D). A significant reduction of LIP overexpression was also observed after treatment with IFNβ (Figure 3C,D, lane 5): this cytokine may affect the CMV promoter under which the cDNA encoding the liver-enriched transcriptional-inhibitory protein (LIP) has been cloned. Either treatment with IFNα or IFNβ and C/EBPβ-LIP expression had little effect on cell viability as measured by MTT assay (Supplementary Figure S2).

### 3.4. Silencing of C/EBPβ-LIP by an Adeno-shRNA Reverses the Inhibitor Effect of LIP on JCPyV Replication

**Figure 4:**
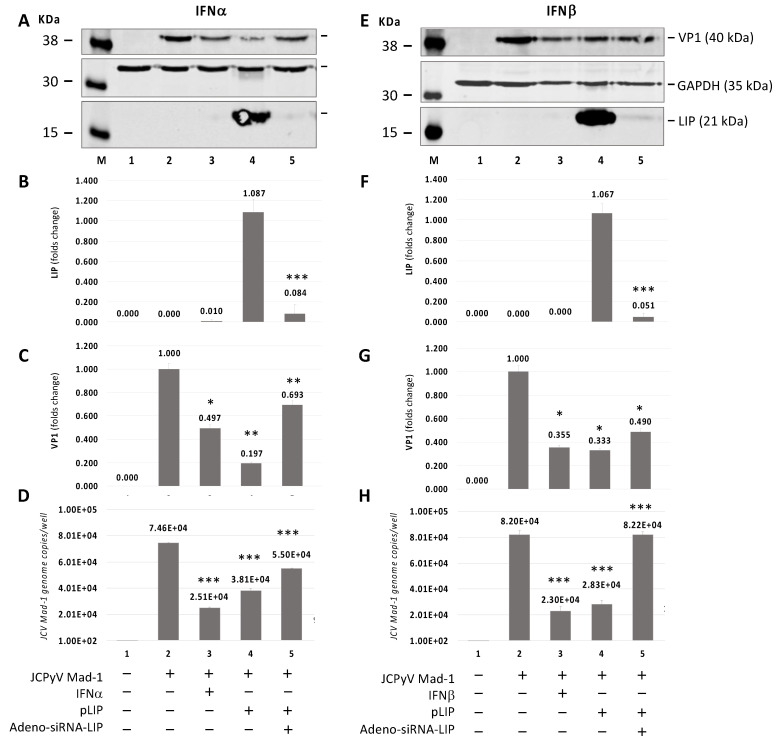
Effect of Adeno-siRNA-LIP on JCPyV-infected SVGA cells transiently transfected with C/EBPβ-LIP expression plasmid in presence or absence of IFNα or IFNβ treatment. Western blot on protein lysis from SVGA cells co-infected with JCPyV Mad-1 at MOI of 1 and Adeno-siRNA-LIP at MOI of 5 after co-transfected with pLIP and treatment with IFNα (**A**–**D**) or IFNβ (**E**–**H**) for 5 days. On the 5th day post-infection (p.i.), the cells were harvested in order to evaluate the expression of LIP (**B**,**F**) and the late capsid protein VP1 (**C**,**G**) by Western blot and to measure the viral load in the supernatant culture media by quantitative PCR (Q-PCR) (D and H). Note: * *p* < 0.05; ** *p* < 0.01; *** *p* < 0.0001.

To investigate the effect of C/EBPβ-LIP on JCPyV infection, we infected SVGA cells with JCPyV or transduced with Adeno-shRNA-LIP or Adeno-null (control) and transfected the cells with C/EBPβ-LIP expression plasmid (Figure 4). JCPyV infection was monitored by Western blot for viral protein VP1 (Figure 4A,C,E,G) and by Q-PCR for viral DNA in the culture supernatants (Figure 4D,H). The Western blot analysis showed a significant reduction of LIP overexpression after Adeno-siRNA-LIP infection (*p* < 0.0001) (Figure 4A,B,E,F, lane 5). A reduction of VP1 expression after the IFNs treatment and Adeno-siRNA-LIP infection has also been observed (Figure 4A,C,E,G, lanes 3 and 4, respectively). On the other hand, the JC viral load in the supernatant detected by Q-PCR was significantly reduced after IFNs treatment and overexpression of LIP (D and H lanes 3 and 4, respectively). These results showed that shRNA depletion of C/EBPβ-LIP reversed the inhibitory effect of LIP expression, indicating a significant role of C/EBPβ-LIP in the JCPyV replication. Either treatment with IFNα or β, C/EBPβ-LIP expression and Adeno-shRNA-LIP and JCPyV infections had little effect on cell viability as measured by MTT assay (Supplementary Figure S3).

### 3.5. Translocation of C/EBPβ-LIP to the Nucleus after IFNα or IFNβ Treatment

**Figure 5:**
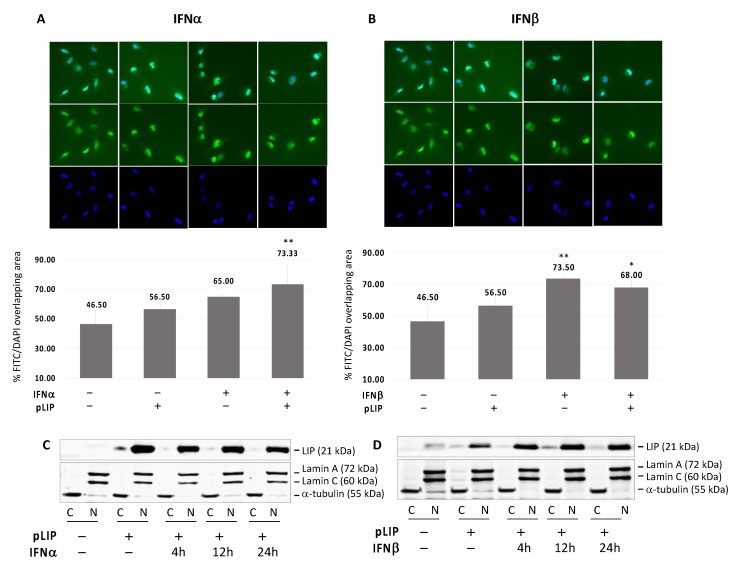
Translocation of C/EBPβ-LIP to the nucleus after IFNα or IFNβ treatment. Immunocytochemistry (ICC) of SVGA cells transiently transfected with pLIP and treated with IFNα (**A**) or IFNβ (**B**) for 24 h. After blocking, the slides were incubated overnight with mouse anti-CEBPβ (H7) antibody (FITC) and then mounted with medium containing DAPI. The ICC images were acquired by BZ-X800 Fluorescence Microscope (KEYENCE Corporation) and analyzed using the dedicated software in order to calculate the percentage of FITC/DAPI merge. Treatment either with IFNα or IFNβ showed a significant overlapping of FITC and DAPI signal (IFNα: *p* ≤ 0.05 and IFNβ: *p* < 0.01) which is an indication of C/EBPβ-LIP enrichment in the nucleus. In order to confirm the nuclear localization of C/EBPβ-LIP after treatment with IFNs, a cell fractionation assay was performed, cells were harvested at corresponding time points (4, 12 and 24 h) after one treatment with IFNα (**C**) or IFNβ (**D**), and the nuclear and cytoplasmic proteins were isolated. Note: * *p* < 0.05; ** *p* < 0.01.

Since we showed that C/EBPβ-LIP inhibits both JCPyV transcription and replication, we preformed immunocytochemistry to investigate subcellular redistribution of C/EBPβ-LIP after IFNα and IFNβ treatment. SVGA cells where transfected with C/EBPβ-LIP, treated with IFNα and IFNβ for 24 h and analyzed by immunocytochemistry for C/EBPβ-LIP. As shown in Figure 5A,B, the distribution of C/EBPβ-LIP was revealed by mouse anti-C/EBPβ (H7) antibody (FITC, green) and nuclear DAPI (blue). As shown, treatment of IFNα and IFNβ led to translocation of C/EBPβ-LIP to the nucleus. Moreover, in order to confirm the nuclear localization of C/EBPβ-LIP after treatment with IFNs, a cell fractionation assay was performed, cells were harvested at corresponding time points (4, 12 and 24 h) after one treatment with IFNα (**C**) or IFNβ (**D**), and the nuclear and cytoplasmic proteins were isolated. As shown, C/EBPβ-LIP is stable in the nucleus and the IFNβ treatment contributes to stabilize the nuclear localization of this protein (Figure 5D).

## 4. Discussion

Interferons (IFNs) are key defenses against viral infections [32]. A variety of viruses can hijack the INFs system, particularly type 1 IFNs, to produce viral proteins that can suppress immune response against virus infection [33]. We have earlier reported LIP isoform as negative regulator of JCPyV transcription from both early and late promoter [34]. LIP isoform of the CCAAT/enhancer binding proteins has been identified as a cellular protein that is upregulated upon ER stress and involved in degradation of polyomavirus JC T-antigen. [16]. IFNα induces inhibitory LIP isoform of C/EBPβ to suppress the HIV LTR in macrophages [18,35]. In the present study, we uncovered a new role of LIP on IFN system on the regulation of JCPyV transcription and replication. Here, we demonstrated that IFNα or β treatment of glial cells induces a dominant negative LIP isoform of C/EBPβ, and this induction of LIP isoform is associated with inhibition both JCPyV viral replication and transcription in our experimental approach.

Luciferase reporter analysis of JCPyV early and late promoter activity in TC620 cells has demonstrated that overexpression of LIP inhibits JCPyV transcription and this effect is more pronounced in the presence of IFNα and IFNβ. Western blot analyses of JCPyV infected SVGA cells showed the downregulation of viral VP1 and JCPyV replication when LIP is overexpressed. This observation was further supported by an experiment that showed that silencing of LIP by shRNA reverses the inhibitory effect of LIP on viral protein expression and replication. This shows that inhibition of JCPyV replication is associated with suppression of JCPyV promoter activity indicating that LIP transcriptional downregulation of viral promoter is one of the mechanisms of attenuation of viral replication. Our observation in this study suggests the existence of the molecular interplay between IFNs type 1 system and C/EBPβ transcription factors that may regulate JCPyV life cycle. Since C/EBPβ isoforms are the predominant transcription factors which are regulated by signal transduction pathways and induced by inflammatory stimuli and C/EBPβ DNA binding element is essential for JCPyV promoter inhibition, IFNα or β treatment induced inhibitory LIP isoform of C/EBPβ is likely responsible for inhibition of both JCPyV replication and transcription. The abrogation of the inhibitory effect of LIP expression on both JCPyV transcription and replication by shRNA targeting LIP strongly support such a conclusion. One level of regulation of C/EBPβ is subcellular localization [15]. Our immunochemistry analysis showed that IFNα or β treatment of SVGA cells overexpressing LIP results in cytoplasm to nucleus relocation of LIP. This further supports the importance of LIP in JCPyV life cycle. It has previously been reported that interferons negatively regulate JCPyV [13,14]. Co et al. (2007) reported that JCPyV replication was significantly inhibited by IFN in primary human fetal glial cells and neutralizing anti-IFN antibody rescued the inhibitory effect of IFN [13]. Assetta et al. [14] found JCPyV infection of primary human renal epithelial cells induced interferon production and activated interferon-stimulated gene expression. In the later study, it was also noted that phosphorylated STAT1 and IFN regulatory factor-9 (IRF9) translocated to the nucleus in JCPyV-infected cells and that blocking the IFNAR and neutralization of IFNα and IFNβ partially relieved inhibition of JCPyV infection [14]. In the present study, we also found that that IFNα or β treatment of glial cells induced STAT1 expression and STAT1 phosphorylation. However, infection of STAT1 knockout clonal cell line with JCPyV in the presence or absence of IFNα suppresses viral replication indicating that IFNα exerts its anti-JCPyV activity via an independent STAT1 induction or activation. Previously, De Simone et al. [36] reported the anti-JCPyV activity of IFNγ. That study has shown that IFNγ inhibits JCPyV replication in glia cells by suppressing T-antigen expression and this downregulation effect is caused by inhibiting translational initiation through a decrease in the phosphorylation of p70, S6K, 4EP1 and AKT. Although the exact mechanism of LIP induction by IFNα or IFNβ is unknown, an earlier study by Dudaronek et al. [35] demonstrated that CUGBP1(RNA binding protein) is required for IFNβ mediated induction of LIP isoform to suppress SIV replication in macrophages. The study by Honda BY et al. [18] has shown an increase in the level of endogenous LIP by IFNα treatment of macrophages, which is associated with repression of HIV LTR. At the present, it is not exactly clear the physiological role of IFNα and IFNβ mediated induction LIP in suppressing JCPyV viral transcription and replication. JCPyV, like other neurotropic viruses, has a latency period; its reactivation leads to PML [37]. However, there are many critical areas where our understanding of JCPyV biology is incomplete. Specifically, the mechanism by which latent JCPyV reactivate to cause PML.

We speculate that the cross talk between IFNs type 1 system and C/EBPβ transcription factors may be essential in controlling the balance of JCPyV latency and reactivation under immunosuppression.

## 5. Conclusions

**Figure 6:**
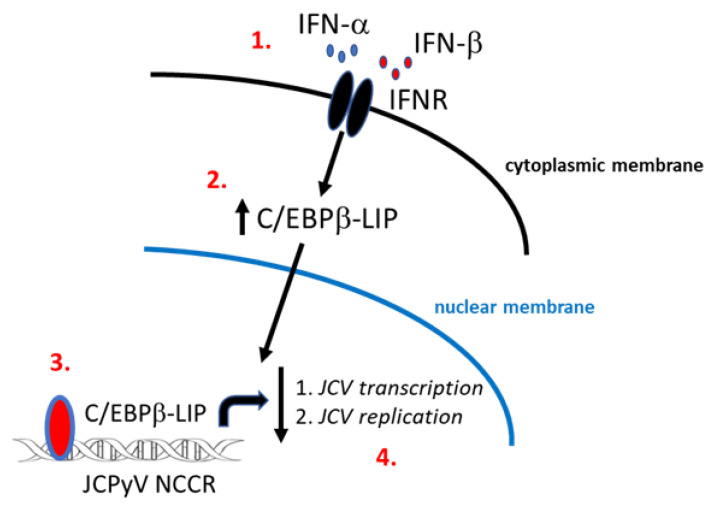
Schematic representation of a novel mechanism of JCPyV transcription and replication control by IFNα and IFNβ by producing a dominate negative isoform LIP of C/EBPβ. IFNα and β binding to IFN receptor (IFNR) activates the signaling pathway which leads to the formation of a phosphorylated STAT1 and STAT2 heterodimer complex to produce transcription factors which binds to IFN-stimulated response elements (ISREs) leading to the production of antiviral effectors [25] (**1.**). Through these signaling pathways IFNs control viral replication, thus, viral spread through the induction of an antiviral state in infected and uninfected cells [26]. In addition to the JAK-STAT pathway, interferons have shown to exert their antiviral activity by modulating the function of some transcriptional factors such as C/EBPβ families [15,27] (**2.**). In this study, the dominant negative isoform LIP of C/EBPβ has been shown to be stable in the nucleus after treatment with IFNα and β, and its interaction with the JCPyV NCCR [10] inhibits JCPyV life cycle (**3.** and **4.**).

In summary, our data presented here provide evidence for a novel mechanism of JCPyV transcription and replication control by IFNα or β by producing a dominate negative isoform of C/EBPβ that inhibits JCPyV life cycle (Figure 6).

## Figures and Tables

**Table 1:** STAT1 e16 and e15 single guide RNAs sequence and primer designed for excision assay.

gRNAs	Nucleotide Positions *	Strand	Sequence (PAM)
STAT1e16	190984331–190984315	Minus	5′-**TGGTTTGGTAATTGACCTCG** AGG-3′
STAT1e15	190985640–190985624	Minus	5′-**TGCTGGCACCAGAACGAATG** AGG-3′
**Oligonucleotides gRNA cloning**	**Nucleotide** positions *	**Strand**	**Sequence**
STAT1e16Fw	-	-	5′-CAC CG**T GGT TTG GTA ATT GAC CTC G**-3′
STAT1e16Rev	-	-	5′-AAA CCG AGG TCA ATT ACC AAA CCA C-3′
STAT1e15Fw	-	-	5′-CAC CG**T GCT GGC ACC AGA ACG AAT G**-3′
STAT1e15Rev	-	-	5′-AAA CCA TTC GTT CTG GTG CCA GCA C-3′
**Primers**	**Nucleotide** Positions *	**Strand**	**Sequence**
STAT1Fw	190984026–190984046	Plus	5′-GGGGAGGGCAGAGCAAAATAA-3′
STAT1Rev	190986115–190986134	Minus	5′-CTGAGGGTATTCAGAGCCGC-3′

* The nucleotide positions are referred to STAT1 ENSG00000115415—RefSeq: NC_000002.12 Homo sapiens chromosome 2, GRCh38.p12 Primary Assembly.

## Data Availability

All the data produced in this article will be available up on request.

## Supplementary Materials

The following are available online at https://www.mdpi.com/article/10.3390/v13101937/s1, Figure S1: Endogenous expression of the three c/EBPβ isoforms (full length (FL), liver activating protein (LAP), and liver inhibitory protein (LIP)) after treatment with IFNα or IFNβ; Figure S2: MTT assay on luciferase assay in order to assess the possible cytotoxic effect of transient transfection and IFNs treatment on the assay outputs; Figure S3: MTT assay on the experiment performed to assess the effect of Adeno-siRNA-LIP on JCPyV-infected SVGA cells transiently transfected with C/EBPβ-LIP expression plasmid, in presence or absence of IFNα or IFNβ treatment, in order to assess the possible cytotoxic effect of transient transfection, IFNs treatment and viral infections on the assay outputs.
