# Supplementary material for: IFNα and β Mediated JCPyV Suppression through C/EBPβ-LIP Isoform
Source: Viruses. 2021 Sep 26;13(10):1937. doi: 10.3390/v13101937 (PMC8537971; doi:10.3390/v13101937)
Supplement: Supplementary file 1 [file viruses-13-01937-s001.zip › viruses-1291265-supplementary.pdf]

viruses

*Supplementary Materials*

# IFNα and β Mediated JCPyV Suppression Through C/EBPβ-LIP Isoform

**Dana May [1,†], Anna Bellizzi [1,†], Workineh Kassa [2], John M. Cipriaso [1], Maurizio Caocci [1] and Hassen S. Wollebo [1,*]**

[1] Department of Neuroscience, Center for Neurovirology—Lewis Katz School of Medicine at Temple University, 3500 N. Broad Street, Philadelphia, PA 19140, USA; tul10237@temple.edu (D.M.); tuf81370@temple.edu (A.B.); john.cipriaso@temple.edu (J.M.C.); tuh20265@temple.edu (M.C.)
[2] Mayo Clinic Hospital and Health Care, 200 First St. S.W. Rochester, MN 55905, USA; etgold4@gmail.com
* Correspondence: siraj123@temple.edu; Tel.: +215-707-7137; Fax: +215-707-4888
† Those authors contributed equally to this work.

**Citation:** May, D.; Bellizzi, A.; Kassa, W.; Cipriaso, J.M.; Caocci, M.; Wollebo, H.S. IFNα and β Mediated JCPyV Suppression Through C/EBPβ-LIP Isoform. *Viruses* **2021**, *13*, 1937. https://doi.org/10.3390/v13101937

Academic Editors: Graciela Andrei and Volker Nickeleit

Received: 23 June 2021
Accepted: 22 September 2021
Published: 26 September 2021

**Publisher's Note:** MDPI stays neutral with regard to jurisdictional claims in published maps and institutional affiliations.

**Copyright:** ©2021 by the authors. Licensee MDPI, Basel, Switzerland. This article is an open access article distributed under the terms and conditions of the Creative Commons Attribution (CC BY) license (http://creativecommons.org/licenses/by/4.0/).

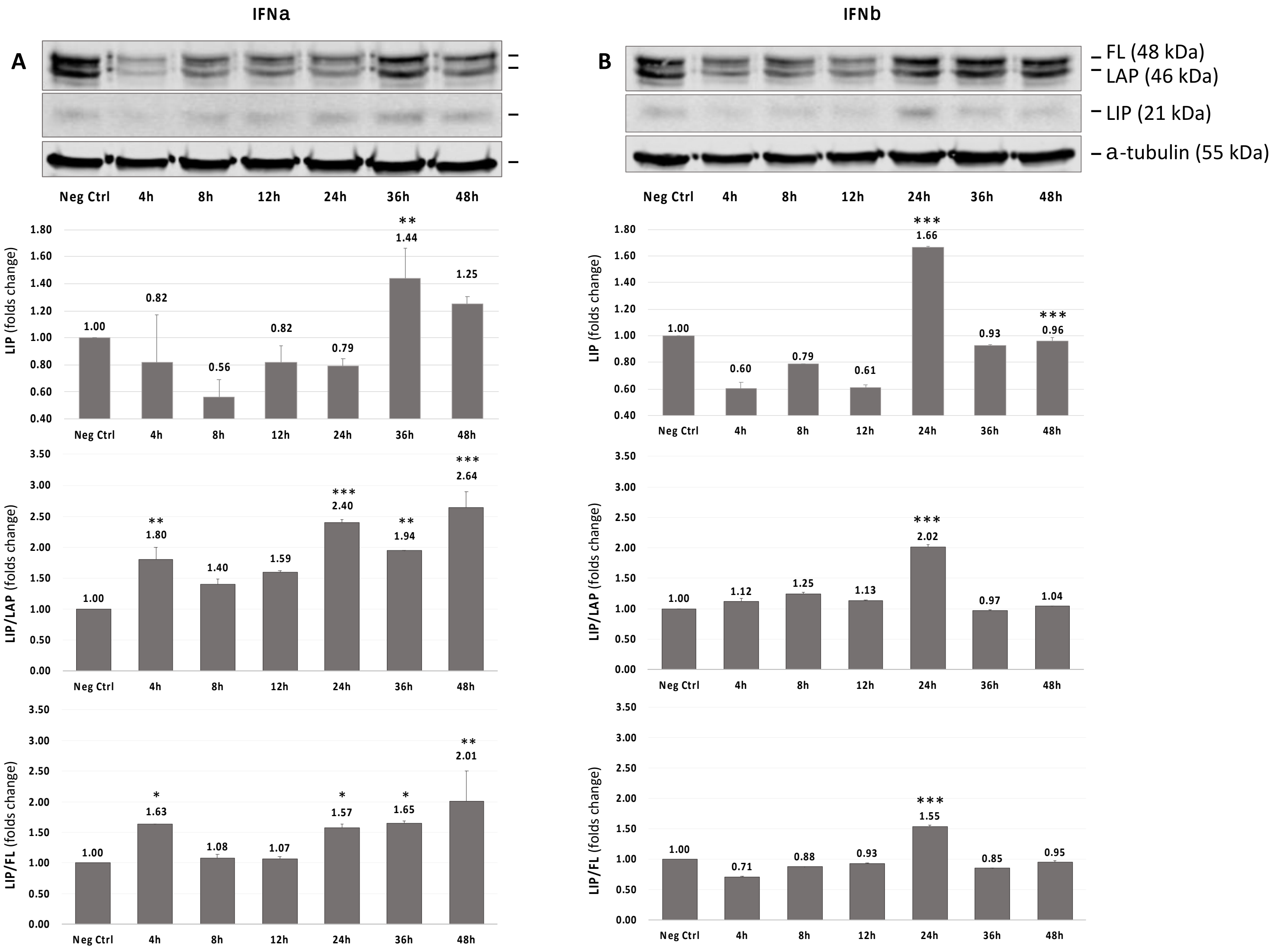


**Figure S1.** Endogenous expression of the three c/EBPβ isoforms [full length (FL), liver activating protein (LAP), and liver inhibitory protein (LIP)] after treatment with IFNα (**A**) or IFNβ (**B**). Western blot on protein lysis from the same experiment reported in Figure 1 (main manuscript). α-tubulin was used as loading control and, for each panel, a densitometry assessment of LIP was also included, using untreated SVGA cells as negative control. Moreover, the ratios between the relative expressions of LIP and the isoforms FL and LAP respectively, were reported. Standard deviation bars are depicted on the graph. p values <0.05 were considered statistically significant. Note: * $p \leqq 0.05$; ** $p < 0.01$; *** $p < 0.0001$.

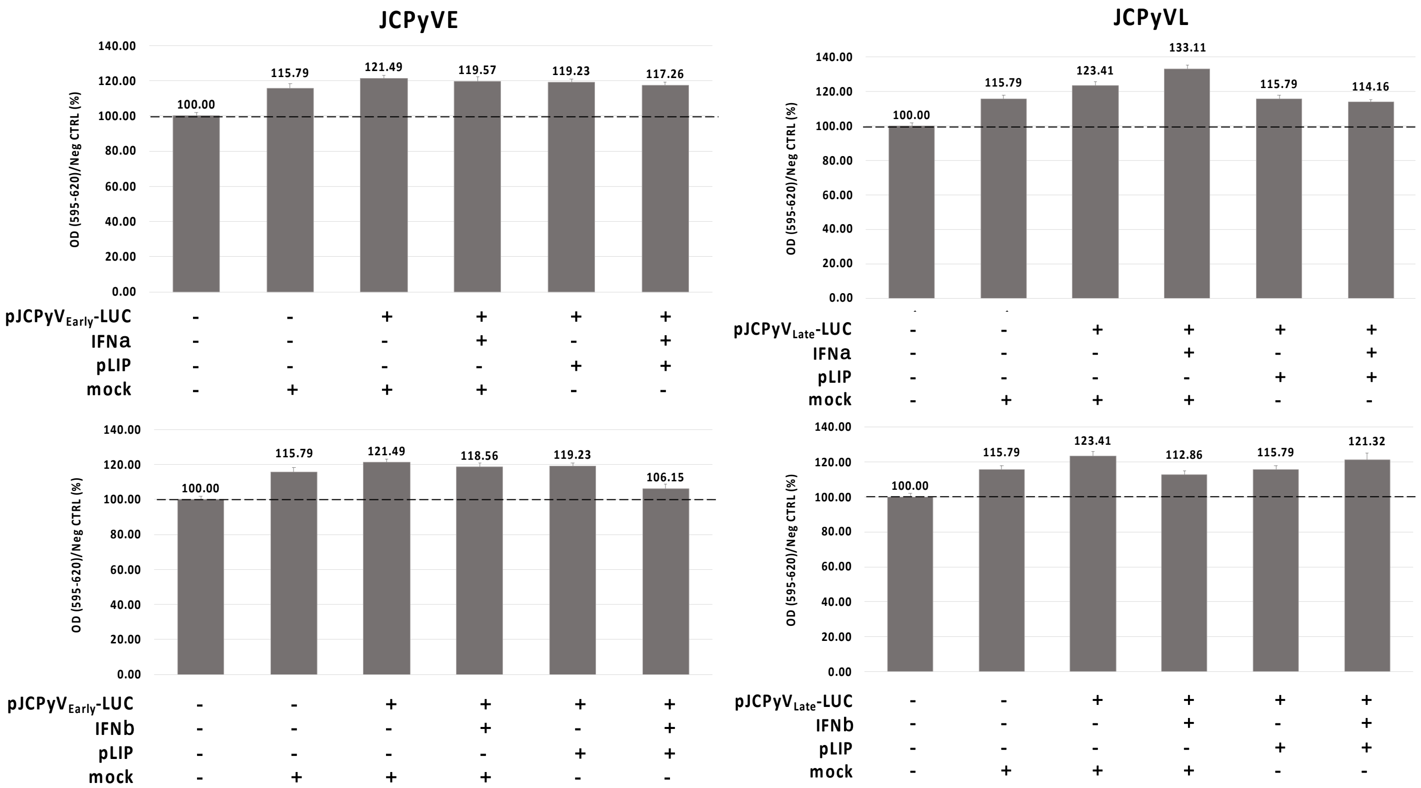


**Figure S2.** MTT assay on luciferase assay in order to assess the possible cytotoxic effect of transient transfection and IFNs treatment on the assay outputs. The co-transfection of pLIP with the reporter plasmid JCPyVE and JCPyVL does not have any effect on TC620 cell viability comparing to the untreated cells both in presence and absence of IFNs treatment.

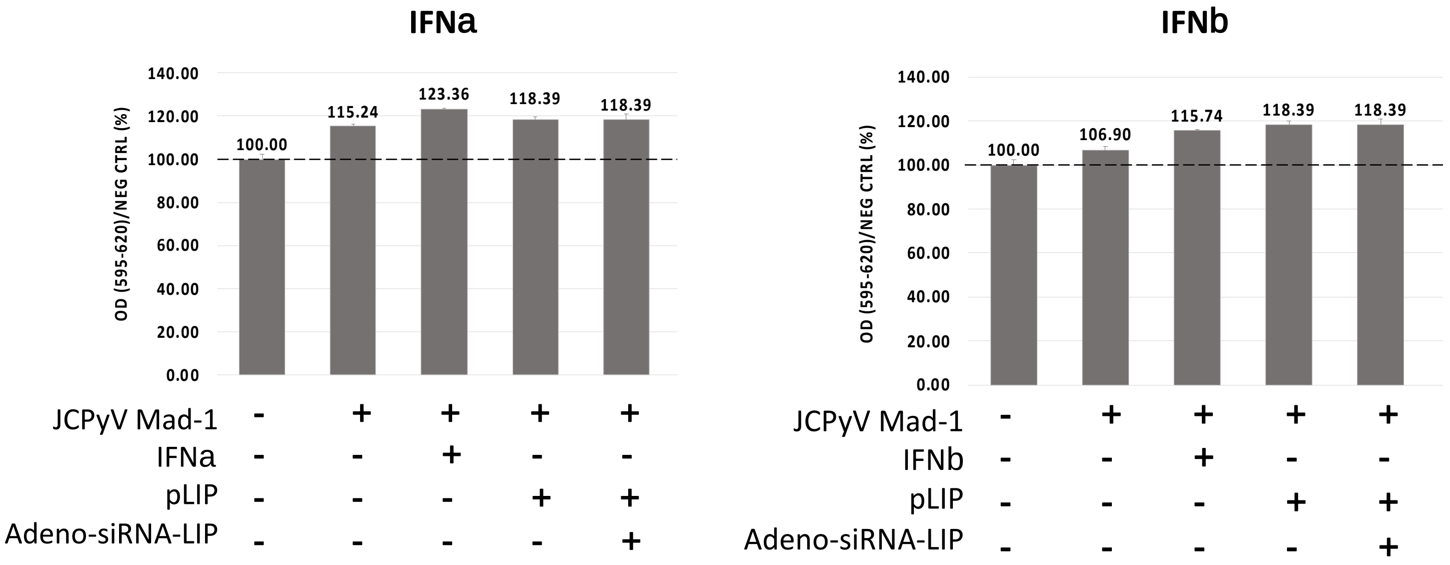


**Figure S3.** MTT assay on the experiment performed to assess the effect of Adeno-siRNA-LIP on JCPyV-infected SVGA cells transiently transfected with C/EBPβ-LIP expression plasmid, in presence or absence of IFNα or IFNβ treatment, to assess the possible cytotoxic effect of transient transfection, IFNs treatment and viral infections on the assay outputs. The co-infection of SVGA cells transiently transfected with pLIP does not have any effect on SVGA cell viability comparing to the untreated cells both in presence and absence of IFNs treatment.
